# Experimental research on compressive strength deterioration of coal seam floor sandstone under the action of acidic mine drainage

**DOI:** 10.1038/s41598-024-55361-6

**Published:** 2024-02-26

**Authors:** Wenmei Han, Zhaoying Chen, Hongtai Liu, Xiang Zheng, Jinwen Wu, Qi Yuan

**Affiliations:** 1 Department of Engineering Mechanics, North University of China, Taiyuan, 030051 China; 2 State Key Laboratory of Coal and CBM Co-Mining, Jincheng, 048012 China

**Keywords:** Coal seam floor sandstone, Acidic mine drainage, Compressive strength, Hydrochemistry, Pore structure, Solid Earth sciences, Energy science and technology, Materials science

## Abstract

In sulphur-coal symbiotic coal seams, after the mining of sulphide iron ore, when the coal resources are mined, the mine water accumulated in the roadway mining area will have a certain impact on the stability of the surrounding rock of the coal seam roadway. Taking the floor sandstone of sulfur coal symbiotic coal seam as the research object, the roof fissure water with pH values of 7.48, 4.81 and 2.62 was used as the experimental solution. 10 experimental schemes were designed to measure the compressive strength of the samples under the action of AMD, and the hydrochemical analysis of AMD was conducted. The pore structures of the samples before and after the action of AMD were analyzed. Based on the hydrochemistry and pore structure, the deterioration mechanism of compressive strength of the coal seam floor sandstone under the action of AMD was explained. The results indicated that the compressive strength of the samples decreased with the increasing action time of AMD. The compressive strength decreased with the increment of the porosity. The concentration of H^+^ ion in AMD was relatively small. Na_2_O in albite dissolved and reacted with water, leading to an increase in the concentration of Na^+^ ion. Soluble substances such as MgCl_2_ and CaSO_4_ in the pore structure dissolved, leading to an increase in the concentration of Ca^2+^ and Mg^2+^ ions. The dissolution of soluble substances and the physical–chemical reactions between solutions and minerals were the essential causes of the continuous deterioration of the compressive strength of the coal seam floor sandstone. The results of this study can provide a theoretical basis for the deterioration of the mechanical properties of the peripheral rock in the roadway of the sulphur coal seam, and can also provide a certain engineering reference for the sulphur coal seam roadway.

## Introduction

The symbiotic sulphur coal seams are widely distributed in China. The pyrite is often found at the bottom of the coal seam, and the development path was arranged along the coal seam. In the 1990s, the symbiotic coal seam of sulfur coal was mainly mined with pyrite, and the coal seam was abandoned as an associated resource^1–3^. Nowadays, the coal resources need to be mined in the symbiotic coal seams of sulfur coal, there are a large number of pyrite development paths and scattered goafs in the coal seam. The phenomenon of AMD accumulation generally exists, which influences the stability of the surrounding rock of the coal seam roadway.

Both domestic and foreign scholars have undertaken studies on the mechanical properties of rocks under the action of mine water. Hu^4^, Guo^5^, Zhou^6^ and Akbari^7^ conducted experimental studies on the microscopic variability of mudstone in the deep coal seam's bottom plate, subjected to infiltration by distilled water, tap water, and mine water. The impact of mine water on the conductivity and disintegration of mudstone surpasses that of distilled water and tap water. Zhang^8^ and Fang^9^ analyzed the water quality, hydrochemical types, and major ions of the underground water silos, and found the hydrological action mainly involved the exchange reaction of cations such as K^+^, Na^+^, Mg^2+^ and Ca^2+^, and the dissolution of carbonate and silicate minerals under the action of mine water's electrical conductivity. Wang^10^ focused on water abundance of aquifers based on the sandstone pore structure. The water abundance and permeability of sandstone aquifers are determined by the pore structure of sandstone. In the same rock layer, the degree of weathering and water-richness of sandstone is positively proportional; in different rock layers, the water-richness of coarse-medium-grained sandstone is greater than that of fine-powdered sandstone. Huo^11^ and Song^12^ conducted experimental studies on the physical properties of sandstone based on the acidic solutions, revealing a lag in the solubility of Ca^2+^ ion during acidic solution infiltration. Zhou^13^, Chen^14^ and Wang^15^ studied the mechanical properties of granite based on alkaline solutions, observing an increase in compressive strength and cohesion of granite with the rising pH of alkaline solutions, and a decrease in the angle of internal friction. Guo^16^ studied the cementing properties of rocks under the action of sulphuric acid solution, noting a decrease in the compressive strength of rocks with decreasing pH. Xu^17^ investigated the mechanical properties of coal under chemical erosion, and found that chemical solution erosion reduced the elastic deformation stage of coal and increased its ductility. Li^18^ and Schimmel^19^ experimentally studied the mechanical properties of the deep underground limestone and quartz sandstone in chemically corrosive environments. The chemical corrosion has a comprehensive and significant impact on the microcrack development in the rock. Peng^20^ analyzed the impact of chemical corrosion on I-type fracture toughness of sandstone using a semicircular bending test, finding that I-type fracture toughness of sandstone initially increased and then gradually decreased with the increase of pH. Parvizpour^21^ studied on the pH effect of sulfuric acid on the physico-mechanical properties of travertine, and found it can affect the physico-mechanical properties of stones and decrease their durability over time. Moghadis^22^ studied on the dissolution and degradation of building stones in sulfuric and nitric acids solutions, and found that the stones with non-carbonate composition are resistant against these acids, while the carbonate ones are not.

In Xicheng mountain ridge edge uplift zone at the southern end of the block depression of Qinshui coal seam, the geological endowment of 15# coal seam in the Taiyuan formation of the carboniferous system is relatively special, with sulphur-coal coexistence, and the thickness of the No. 15 coal seam ranges from 1.3 to 3.6 m. Sulphurous iron ore is deposited in the bottom plate of No. 15 coal seam, which is sandstone or sulphide-bearing sandstone with disseminated, oolitic or nodular sulphide, with an average thickness of 1.0 m, and the mine water in the area is generally acidic. This study will analyze the hydrochemical characteristics of the mine water and investigate the compressive strength of sandstone in the bottom of symbiotic coal seams under the action of mine water. The study aims to identify the variation pattern of compressive strength of sandstone of the sulphur-coal syngenetic coal seam bottom plate over time, further analyzing the changing mechanism of compressive strength of sandstone based on the pore structure. The findings of this study will provide valuable insights into the stability of the surrounding rocks in sulphur-coal coeval seam roadways.

## Experimental research

### Experimental samples

**Figure 1:**
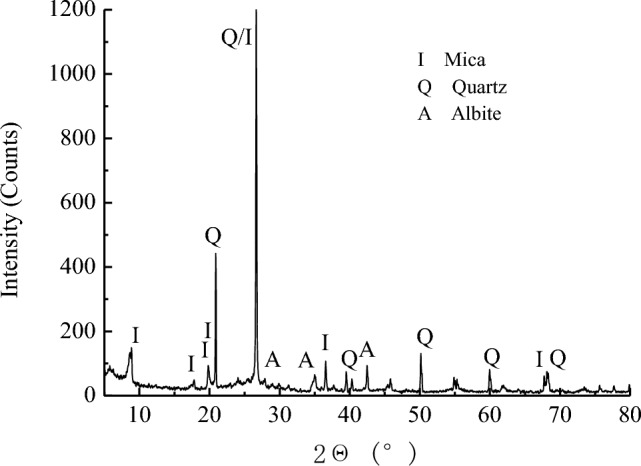
Diagram of diffraction X-ray of the sample.

The sandstone samples used in the experiment were collected from the 15,108 working face of Huiyang mine in Yangcheng, Shanxi. It belongs to the Shanxi Formation of the Lower Permian, and is a terrestrial detrital sedimentary rock. The mine was originally a coal-based sulphide mine, mining sulphur resources, and the resources were consolidated into a coal mine, mining the No. 15 coal seam, with sandstone and sulphide underlays. The samples of collected sandstone were sealed downhole. The core specimens were machined into cylinders of Φ50 × 100 mm using a rock cutter and rock drilling machine, and the quantity was 10. The structural integrity and uniformity of the rocks seem good, with no laminations or cracks. The specimen was characterised by X-ray diffraction (XRD) spectroscopy, and the XRD pattern of the specimen was shown in Fig. 1.

### Experimental apparatus

The hydrochemical characteristics of AMD were analyzed both before and after the action of AMD by Atomic Absorption Spectrophotometer (AAS), with an operating wavelength range is 190-900nm, a hollow cathode light source, square wave pulse power supply, an atomiser of the hybrid type with a length of 100 mm, a Single-slit burner, Cheney-Ternay grating monochromator beam splitter system with 1800 lines/mm of grating scribing. To test the anions in AMD through suppressed conductivity, a CIC-D100 ion chromatograph was employed. The drenching solution used was comprised of 3.6 mM Na_2_CO_3_ + 4.5 mM Na_2_HCO_3_, and the pH range of the solution spanned from 0 to 14.

The pore structure of the samples was analyzed. A µCT225kVFCB type high precision micro-CT experimental system was used, with the maximum diameter of the samples scanned by the X-ray imaging system up to 50mm. The magnification capability reached up to 400 times, achieving a resolution as fine as 0.5 µm. The generation of binary images of the test rock samples was digitally image processed, and generated cross-sectional images spanning 300–1400 layers.

### Hydrochemical analysis of AMD

AMD was collected from three locations: the return airway 885 m, the haulage gateway 190 m and 100 m points in the 15,108 working face.

**Figure 2:**
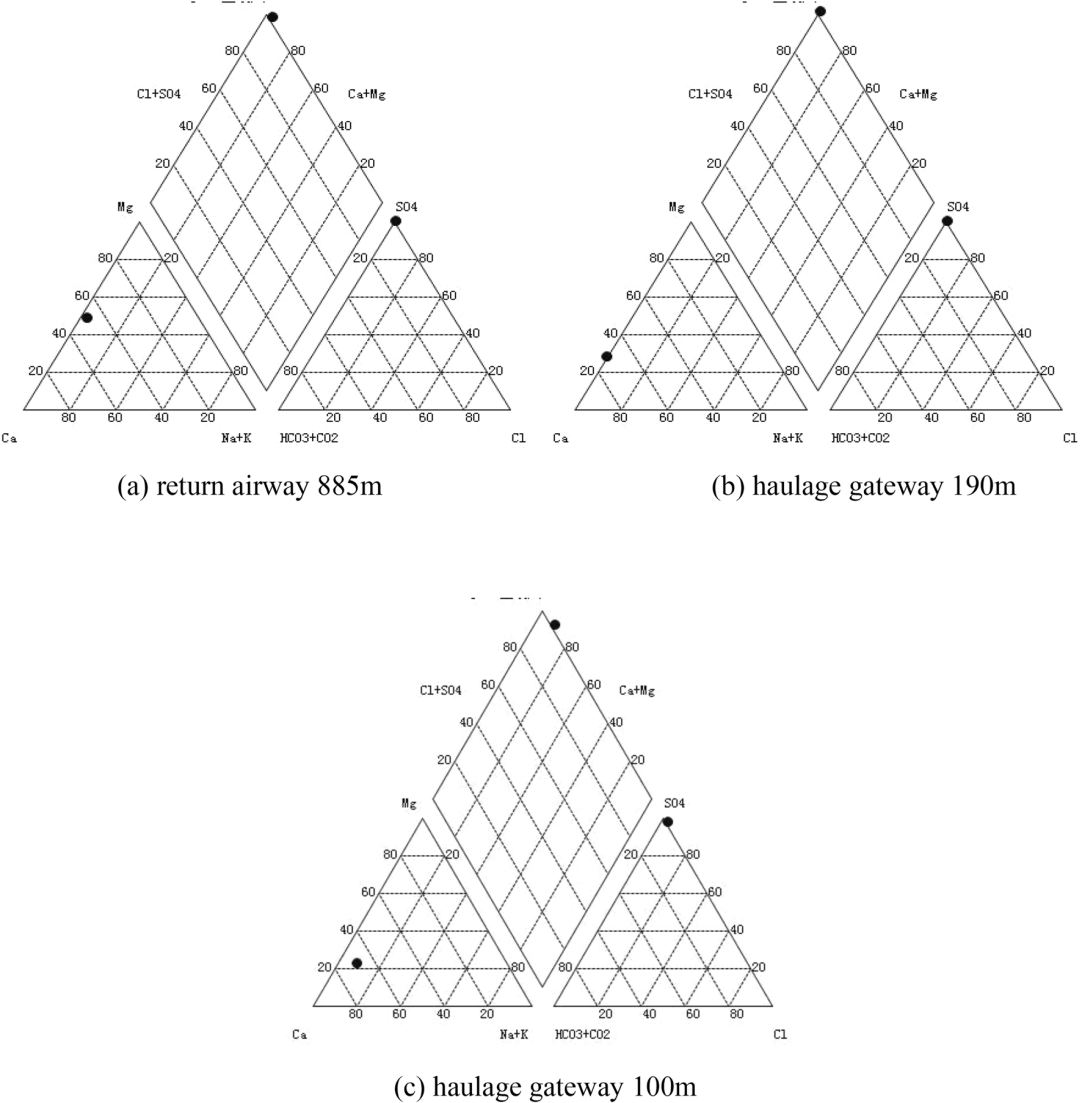
Piper diagrams of the hydrochemical characteristics of AMD.

The hydrochemical characteristics of AMD were analyzed. Figure 2 depicts Piper diagrams of the hydrochemical characteristics of AMD.

The piper trilinear diagram is a common graphical method of indicating hydrochemical ion composition. The diagram is composed of three parts, each edge representing equal parts of 100. The bottom-left triangle denotes the relative content of Ca^2+^, Mg^2+^, and Na^+^ ions, while the bottom-right triangle indicates the relative content of Cl^−^, SO_4_^2−^, and HCO_3_^−^ + CO_2_^2−^ ions. The intersection point, derived from the extension of the rhombus towards the top, indicates the relative content of anions and cations in AMD, expressed as a percentage of milligrams equivalents per liter. The diagram can visually display the hydrochemical characteristics of AMD in general. Table 1 shows the hydrochemical characteristics of AMD.

**Table 1:** Hydrochemical characteristics of AMD.

Sampling location	pH	Ca^2+^ mg/L	Mg^2+^ mg/L	Na^+^ mg/L	Cl^−^ mg/L	SO_4_^2−^ mg/L
Return airway 885 m	7.48	47.3	7.6	8.7	22.9	645.7
Haulage gateway 190 m	4.81	637.2	148.6	10.4	14.6	7270.9
Haulage gateway 100 m	2.62	343.2	208.1	39.1	25.6	8007.7

### Experimental schemes

In the actual project, the sandstones on the coal seam bottom are located in different positions. The hydrochemical composition of AMD and its infiltration time of the bottom sandstone are different, so it is not possible to investigate them one by one. In order to investigate the compressive strength of the samples subjected to different hydrochemical solutions, 10 experimental schemes were designed. Scheme 1 was used to investigate the compressive strength of the original sample. Schemes 2–4 were used to investigate the compressive strength under the action of AMD with a pH value of 7.48 and infiltration time of 10, 20, and 30 days, respectively. Schemes 5–7 were used to investigate the compressive strength under the action of AMD with a pH value of 4.81, with infiltration time of 10, 20, and 30 days, respectively. Schemes 8–10 were used to investigate the compressive strength under the action of AMD with a pH value of 2.62, with infiltration time of 10, 20 and 30 days, respectively.

## Experimental results and analysis

### Compressive strength of samples under AMD

**Figure 3:**
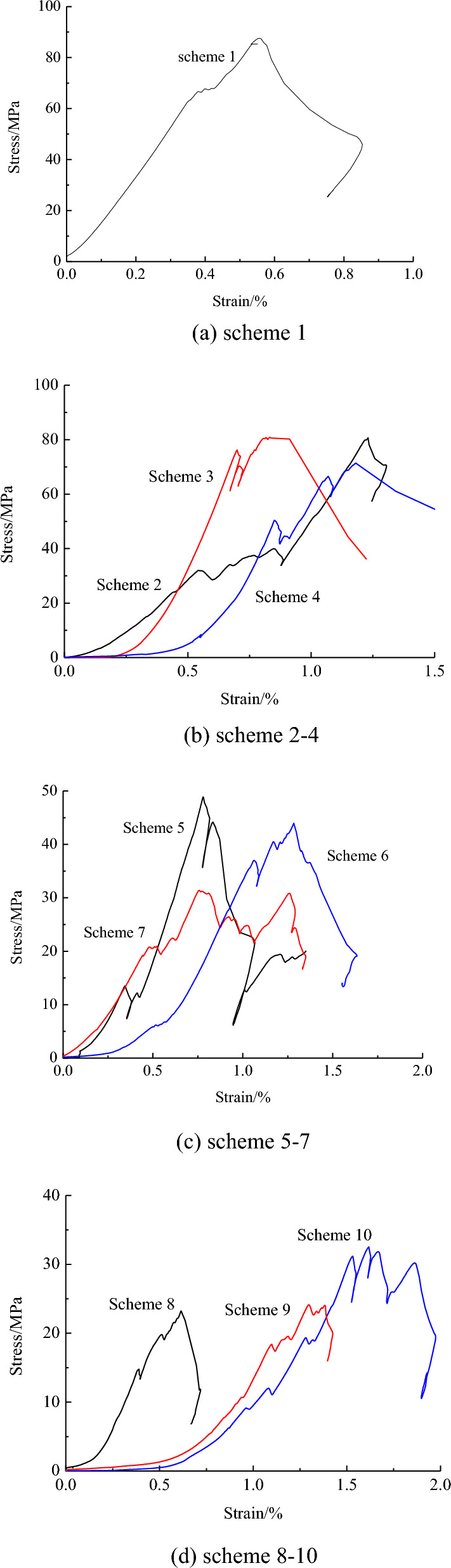
Stress–strain curves of the samples.

The compressive strength of the samples was tested using the microcomputer-controlled electro-hydraulic servo testing machine WDW-200D. Figure 3 shows the stress–strain curves of the samples subjected to AMD.

Under the action of AMD with different pH values and different infiltration time, the stress–strain curves of the samples basically exhibit three stages: the crack compaction and closure stage, elastic deformation stage, and failure stage. Due to the inherent brittleness of sandstone, it presents typical characteristics of fracture damage observed in brittle materials, and the stress reaches the peak value and then decreases sharply. In scheme 1, the compressive strength of the original sample was 87.56 MPa. In schemes 2–4, the compressive strength was 80.72 MPa, 80.86 MPa, and 71.4 MPa, respectively, which reduced by 7.65–18.46%. In schemes 5–7, the compressive strength was 48.89 MPa, 44.0 MPa, and 31.42 MPa respectively, which reduced by 44.2–64.12%. In schemes 8–10, the compressive strength exhibited a significant decrease, measuring 32.52 MPa, 27.94 MPa, and 23.2 MPa, respectively, with reductions ranging from 62.86 to 73.50%.

**Figure 4:**
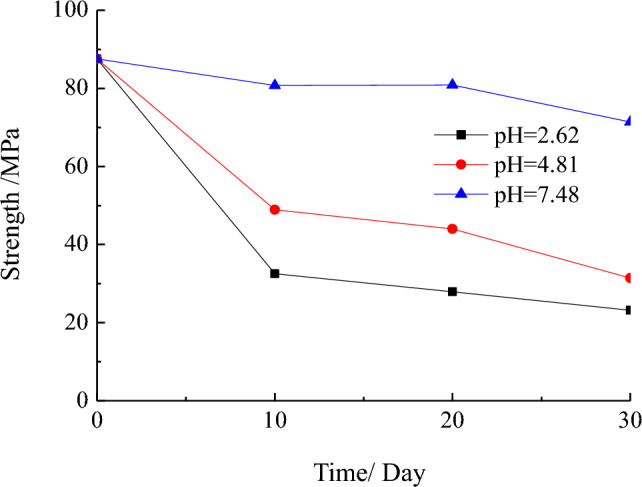
Curve between the compressive strength of the samples and the infiltration time.

Figure 4 shows the variation curves of compressive strength of test samples with different infiltration time under the action of AMD with different pH values.

Under the action of AMD, the compressive strength of the samples decreases with increasing infiltration time, albeit the decrease amplitude is relatively small, not exceeding 12%. As the acidity of the AMD increases, the compressive strength of the samples rapidly decreases. When the pH value is 2.62, the strength has decreased by up to 73.5% compared with the original sample. This underscores the substantial deteriorating impact of AMD on the compressive strength of sandstone at the coal seam bottom, with pH values of AMD emerging as the primary factor contributing to a rapid reduction in sandstone compressive strength.

### Compressive strength of the samples based on the pore structure

**Figure 5:**
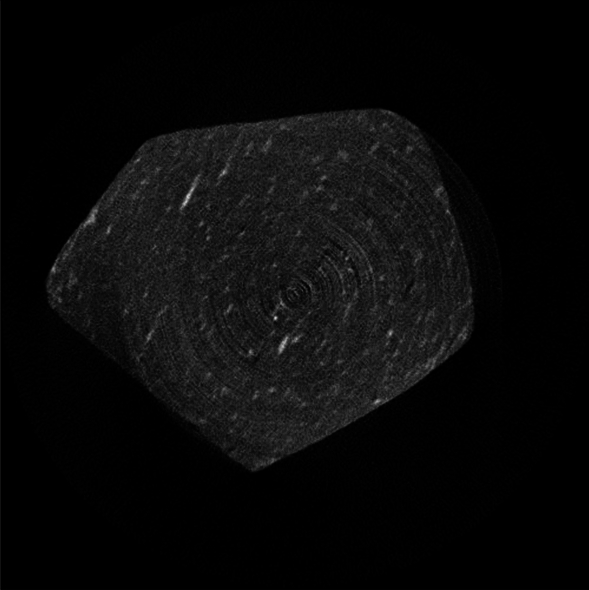
CT single-slice cross-sectional image of the sample in scheme 1.

The combination of µCT, digital image processing, and three-dimensional reconstruction is a new, simple, and feasible method for analyzing the pore structures of rocks. A single digital image was processed through image segmentation, binarisation, and compression, generating new images with varying resolutions. By considering the pixel size of the new image as the pore aperture, the rule governing the variation in rock porosity with changes in pore aperture was estimated from the µCT images. The scanned samples, with a length of 15 mm and a diameter range of 1.5–2.0 mm, allowed for the segmentation of the maximum statistical area from the µCT images with Matlab. The statistical area of slice 100 is shown in Fig. 5.

**Figure 6:**
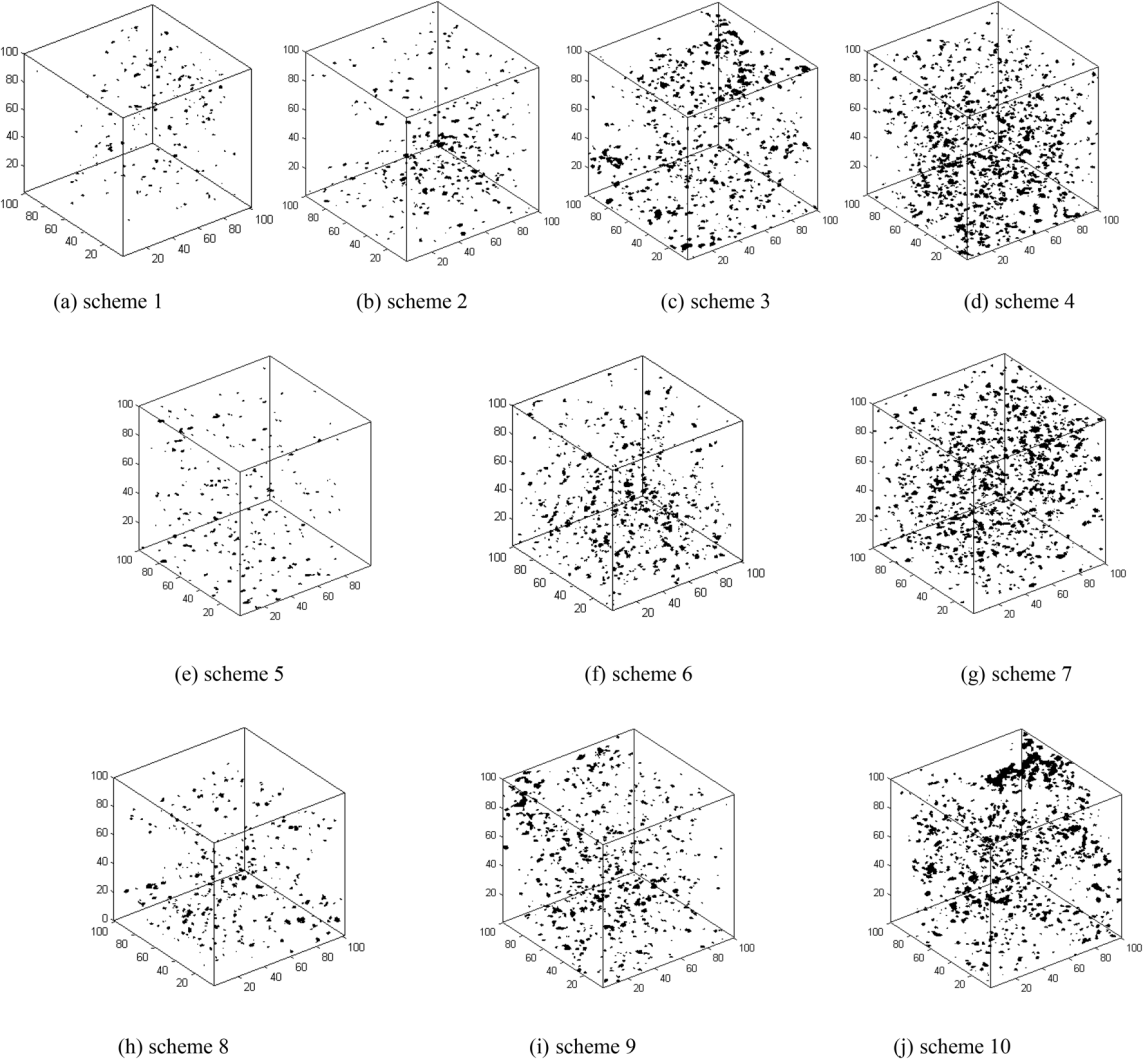
Three-dimensional digital images of the samples.

The pixel matrices of the single-layer images are 2048 × 2048, with a scanning unit size of 1.47 μm, resolving pores with a diameter of 1.47 μm. The segmentation of 100-layer profile images was performed with a segmentation area of 100 × 100 pixels, creating a three-dimensional digital image through the volume rendering algorithm of visualized reconstruction^19^. The three-dimensional digital image of pore structure is presented in Fig. 6.

In Fig. 6, the units of the three coordinate axes are pixels, ranging from 0 to 100 pixels. Figure 6a displays a three-dimensional digital image of the pore structure of the original sample, with a calculated porosity of 3.78%. Figure 6b–d exhibit three-dimensional digital images of the pore distribution for test rock samples in schemes 2 to 4 under the action of AMD with a pH value of 7.48 and infiltration time of 10, 20, and 30 days. The porosity is 3.85%, 5.57%, and 7.43% in order. Figure 6e–g demonstrate, under the action of AMD with a pH value of 4.81 and different infiltration time, porosities of 4.12%, 6.63%, and 8.74% for test rock samples, respectively. Figure 6h–j depict, under the action of AMD with a pH value of 2.62 and varying infiltration time, porosities of 4.63%, 6.88%, and 9.16%, respectively.

**Figure 7:**
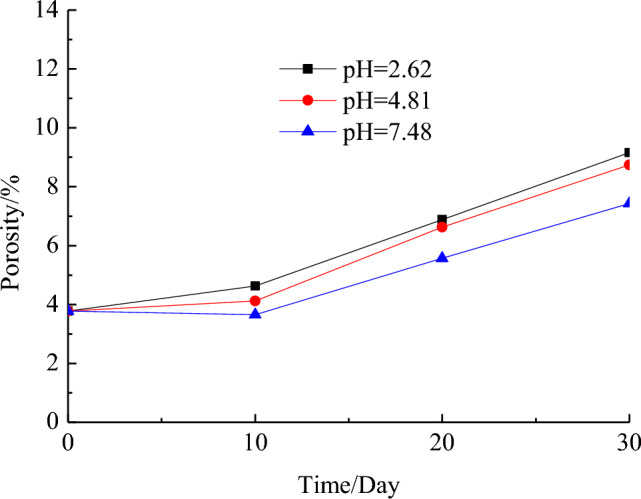
Curve between the porosity of the samples and the infiltration time under AMD.

**Figure 8:**
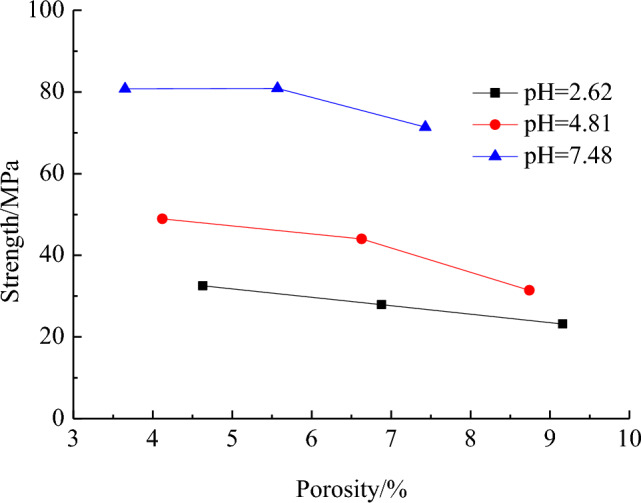
Curve between the compressive strength of the samples and porosity under AMD.

Figure 7 shows the variation curve of porosity for test rock samples over the duration of mine water action when the discernible pore size is 1.47 μm. For pH values of 7.48, 4.81, and 2.62, respectively, the porosity of the samples demonstrates an increasing trend with an increase in the acidity of AMD and infiltration time. Figure 8 presents the curves depicting the relationship between the compressive strength of the samples and porosity under the action of AMD with pH values of 7.48, 4.81, and 2.62, all displaying a decreasing trend with an increase in porosity.

### Deterioration mechanism analysis of the compressive strength

The sandstone samples were collected from 15# coal seam bottom. The AMD generated by the fissure water of the roof plate exhibited generally acidic properties. Consequently, the surface of the samples showed signs of erosion, acquiring a rusty coloration. One of the X-ray diffraction patterns of the samples is depicted in Fig. 1, and the mineral composition of the sample was quantitatively analyzed using an adiabatic method. The sample's mineral composition includes 45% mica, 35% quartz, and 20% albite. The chemical composition of mica is predominantly composed of SiO2 and Al2O3. Quartz is primarily composed of SiO_2_, and the chemical composition of albite is mainly Na_2_O, SiO_2_, and Al_2_O_3_. To investigate the deterioration of the samples under the action of AMD, the hydrochemical characteristics of schemes 2–10 were analyzed. The results of hydrochemical characteristics are shown in Table 2.

**Table 2:** Hydrochemical characteristics of AMD.

Scheme	Ca^2+^ mg/L	Mg^2+^ mg/L	Na^+^ mg/L	Cl^−^ mg/L	SO_4_^2−^ mg/L
2	176.0	28.5	14.0	19.6	429.5
3	126.0	45.5	15.3	16.9	399.0
4	177.0	33.4	16.6	22.1	454.0
5	885.1	130.0	19.2	13.8	7198.0
6	833.9	132.0	21.3	21.3	7775.0
7	844.6	181.8	33.9	18.6	6635.0
8	625.8	238.0	51.7	13.0	7800.0
9	614.0	230.0	60.6	25.9	7255.3
10	655.0	235.5	60.5	17.8	7655.5

Under the action of AMD with a pH value of 2.62 and different infiltration time, the concentration of cations Ca^2+^ and Mg^2+^ increased compared to AMD that had not yet acted on the specimens in Table 1. After 10, 20, and 30 days of the mine water's action on the samples, the concentration of Ca^2+^ ions increased by 282.6, 270.8, and 311.8 mg/L respectively, with the percentage increase being 82.3%, 78.9%, and 90.8%, respectively. The concentration of Mg^2+^ increased by 29.9, 21.9, and 27.4 mg/L, with the percentage increase being 14.4%, 10.5%, and 13.2%, respectively. Under the action of AMD with pH values of 4.81 and 7.48, the concentration of Ca^2+^ and Mg^2+^ cations exhibited variations, ranging from 196.7 to 247.9 and − 18.6 to 33.2, 78.7–129.7, and 5.3–7.9 mg/L, respectively. Considering the reasons for these variations, the experimental sandstone samples were collected from the floor of the No. 15 coal seam, which was a sedimentary rock coexisting with sulfur coal. The overall structure of the sedimentary rock is dense and uniform, containing a certain amount of soluble minerals. After AMD acted on the samples, the soluble substances MgCl_2_ and CaSO_4_ in the samples dissolved, leading to an increase in the concentration of Ca^2+^ and Mg^2+^ ions.

When the pH values of AMD were 7.48, 4.81, and 2.62, the concentration of H^+^ ions was relatively low. The Si^4+^ and Al^3+^ ions in the samples existed in the form of silicate and aluminate, respectively, with relatively low concentrations of Si4+ and Al3+ ions in the AMD solution. Na2O in albite would dissolve, existing in the form of Na+ ions, as indicated by the reaction equation.

1 $$ {\text{Na}}_{{\text{2}}} {\text{O}} + {\text{H}}_{{\text{2}}} {\text{O}} = {\text{2NaOH}} $$

As a result, after AMD acted on the samples for different durations, the concentration of Na^+^ ions in the AMD solution increased. From Table 2, it can be observed that after AMD with different pH values acted on the samples for increasing duration, the concentration of Na^+^ ions increased by 12.6–21.5 mg/L, 19.2–21.3 mg/L, and 5.3–7.9 mg/L, respectively. Under conditions where the pH values are similar, the Cl^−^ and SO_4_^2−^ ions generate basic magnesium chloride such as Mg(OH)Cl and Ca_2_(OH)_2_SO_4_, resulting in a decrease in their concentration after some experimental reactions. Taking Mg(OH)_2_ as an example, when the pH is 6.75, the concentration of Mg^2+^ ions is 33.4 mg/L.

2 $$ {\text{pOH}} = {\text{14}} - {\text{6}}.{\text{75}} = {\text{7}}.{\text{25}} $$

3 $$ {\text{C}}\left( {{\text{OH}}^{ - } } \right) = {\text{1}}0^{{ - {\text{7}}.{\text{25}}}}  $$

4 $$ {\text{Q}} = {\text{C}}^{{\text{2}}} \left( {{\text{OH}}^{ - } } \right) \times {\text{C}}\left( {{\text{Mg}}^{{{\text{2}} + }} } \right) $$

This demonstrates that Ca^2+^ and Mg^2+^ exist in the form of ions.

In these equations, pOH is the pH value of OH^−^; C is the concentration, mol/L; Q is the ionic product of OH^−^ and Mg^2+^, which is dimensionless; Ksp is the capacity product, which is also dimensionless; M is Molar mass of Mg^2+^ ions, taken as 24 g/mol. Both Ca^2+^ and Mg^2+^ exist in the form of ions. The relationship illustrated can be expressed as follows.

$$ \begin{aligned}    & {\text{Q}} = (10^{{ - 7.25}} )^{2}  \times 33.4/24 = 1.39 \times 10^{{ - 17.5}}  \\     & {\text{Ksp}} = 5.61 \times 10^{{ - 12}}  \\     & {\text{Q}} < {\text{Ksp}} \\  \end{aligned}  $$

After the action of AMD on the samples, the dissolution of soluble substances such as MgCl_2_ and CaSO_4_ led to the variation in the pore structure of the samples. This alteration in pore structure distribution can be clearly observed from Fig. 5, and Fig. 6 indicates that the porosity has increased to varying degrees. As the infiltration time increases, the porosity of the samples shows an increasing trend. As the porosity increases, the compressive strength of the samples shows a decreasing trend. The dissolution of soluble substances and the physical–chemical reactions between solutions and minerals lead to the generation of new microcracks within the sandstone and the propagation of pre-existing microcracks. The continuous deterioration of the compressive strength of the samples is a macroscopic consequence of the gradual expansion and penetration process of the internal cracks within the sandstone.

## Results

The research focused on the sandstone from the floor of 15# coal seam, coexisting with sulfur coal. X-ray diffraction analysis was conducted to examine its mineral composition. The AMD which the pH values are 7.48, 4.81, and 2.62 was collected, and hydrochemical characteristics testing was performed. AMD samples, characterized by pH values of 7.48, 4.81, and 2.62, were collected for hydrochemical characteristics analysis. 10 experimental schemes were designed, and the stress–strain curves of the compressive strength of the samples were analyzed under the action of AMD with different pH values and infiltration durations. This analysis revealed the variation pattern of the compressive strength of the sandstone from the coal seam floor subjected to AMD. Furthermore, the study provided an explanation of the mechanism governing the deterioration of compressive strength in the sandstone, drawing insights from both hydrochemistry and pore structure analyses.

1. The samples were collected from the floor sandstone coexisting with sulfur and iron mines. AMD derived from the fissure water in the roof displayed an overall acidic nature, resulting in the surface of the sandstone samples eroded a rusty color. Based on the X-ray diffraction spectrum, the mineralogical components of the sample were mainly mica, quartz, and albite, with chemical compositions primarily composed of Na_2_O, SiO_2_, and Al_2_O_3_.
2. The compressive strength of the original sample was 87.56 MPa. Under the action of AMD with a pH value of 7.48, the compressive strength of the samples exhibited a reduction of no more than 12%. Notably, under the action of AMD with a pH value of 2.62, the maximum decline in compressive strength reached 73.5%.
3. The samples were scanned with µCT, and the three-dimensional digital images depicting the pore structures of samples were reconstructed utilizing Matlab language. When the distinguished pore diameter is 1.47 μm, the porosity of the samples showed an increasing trend with the increase of AMD acidity and infiltration time, and the compressive strength of the samples decreased with the increment of the porosity.
4. The hydrochemical characteristics were analyzed on the AMD treated with the samples. The concentration of anions including H^+^, Si^4+^ and Al^3+^, exhibited a reduction, while Na_2_O in albite dissolved and manifested in the form of Na^+^ ion. The dissolution of soluble substances such as MgCl_2_ and CaSO_4_ within the pore structure led to an increase in the concentration of Ca^2+^ and Mg^2+^ ions, concomitant with an increase in porosity. The dissolution of soluble substances and the physical–chemical reactions between solutions and minerals led to the generation of new microcracks within the sandstone, further propagating the original microcracks. The continuous deterioration of the compressive strength of the samples was a macroscopic consequence of the gradual propagation and penetration process of internal cracks in sandstone.

## Data Availability

The authors confirm that the data supporting the findings of this study are available within the manuscript and its additional files.
